# Cumulative Dialytic Glucose Exposure is a Risk Factor for Peritoneal Fibrosis and Angiogenesis in Pediatric Patients Undergoing Peritoneal Dialysis Using Neutral-pH Fluids

**DOI:** 10.1016/j.ekir.2022.08.013

**Published:** 2022-09-03

**Authors:** Yoko Shirai, Kenichiro Miura, Takeshi Ike, Kensuke Sasaki, Kiyonobu Ishizuka, Shigeru Horita, Sekiko Taneda, Daishi Hirano, Kazuho Honda, Yutaka Yamaguchi, Takao Masaki, Motoshi Hattori

**Affiliations:** 1 Department of Pediatric Nephrology, Tokyo Women’s Medical University, Tokyo, Japan; 2 Department of Nephrology, Hiroshima University Hospital, Hiroshima, Japan; 3 Department of Pathology, Kidney Center, Tokyo Women’s Medical University, Tokyo, Japan; 4 Department of Pathology, Tokyo Women's Medical University, Tokyo, Japan; 5 Department of Pediatrics, The Jikei University School of Medicine, Tokyo, Japan; 6 Department of Anatomy, Showa University School of Medicine, Tokyo, Japan; 7 Yamaguchi Pathology Laboratory, Chiba, Japan

**Keywords:** fibrosis, pediatric nephrology, peritoneal dialysis, peritoneal membrane

## Abstract

**Introduction:**

Neutral-pH dialysate has been reported to be beneficial to prevent the peritoneal pathological changes in adult peritoneal dialysis (PD) patients, but its use is controversial in pediatric PD patients. In addition, the impact of cumulative dialytic glucose exposure has not been examined.

**Methods:**

Pediatric PD patients using conventional fluids (conventional group, *n* = 31) or those using neutral-pH fluids (neutral-pH group, *n* = 33) were compared. Clinical risk factors for peritoneal pathological changes in the neutral-pH group were analyzed using generalized linear modeling. Furthermore, the mechanisms of peritoneal pathological changes were explored using immunohistochemical studies and cultured cells.

**Results:**

The median (interquartile range) duration of dialysis was 3.2 (1.7–5.3) years in overall patients. After propensity score matching, the conventional group showed increased thickening of the submesothelial compact (SMC) zone and lower luminal-to-vessel diameter (L/V) ratio than the neutral-pH group. In the neutral-pH group, the cumulative dialytic glucose exposure was an independent risk factor for greater thickness of the SMC zone (odds ratio [OR], 1.54; 95% confidence interval [CI], 1.16–2.05) and higher submesothelial microvessel density (OR, 1.29; 95% CI, 1.01–1.64). Immunohistochemical study showed that cumulative dialytic glucose exposure correlated with the proportion of the tissue expressing hypoxia inducible factor -1α (HIF-1α) and vascular endothelial growth factor-α (VEGF-α). In human peritoneal mesothelial cells, high glucose significantly increased HIF-1α and VEGF-α expressions.

**Conclusion:**

Cumulative dialytic glucose exposure is an independent risk factor for peritoneal fibrosis and angiogenesis in pediatric patients undergoing PD using neutral-pH fluids, which might be associated with greater VEGF-α production by myofibroblasts implying a hypoxic response.

## Graphical abstract

**Figure:**
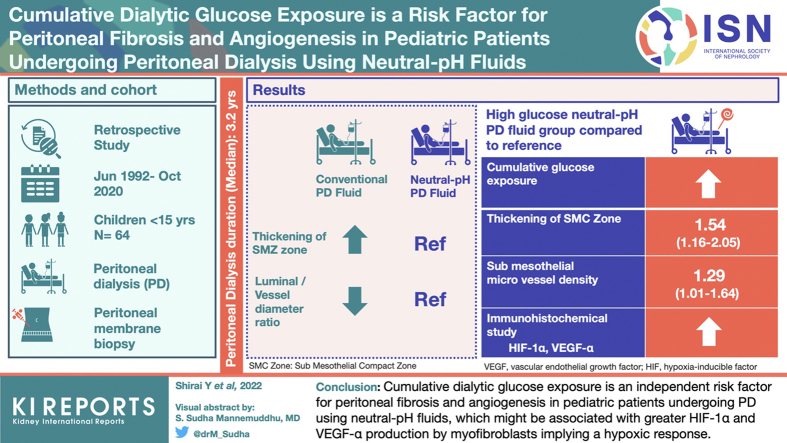

PD is widely used for the treatment of kidney failure in children, and glucose is the most common osmotically active substance used in dialysates, which induce ultrafiltration through the peritoneal membrane.^1^ PD provides continuous therapy, and thus acts more like the kidney than hemodialysis. In addition, it is compatible with fewer dietary restrictions. Nevertheless, it has disadvantages, such as the potential for infection at the exit site, peritonitis, and encapsulating peritoneal sclerosis,^2^ which is a severe complication associated with long-term use of PD.^3^

The pathological findings associated with encapsulating peritoneal sclerosis in patients who are using conventional acidic fluids include expansion of the SMC zone, with varying degrees of hyalinosis, and vasculopathies of postcapillary venules, characterized by luminal narrowing and new membrane formation.^4^^,^^5^ Honda *et al.* ^6^ showed that the L/V diameter ratio of postcapillary venules is a useful histologic means of quantifying the severity of the vasculopathy in adults undergoing PD. Conventional fluids are a source of glucose degradation products (GDPs), which are generated during heat sterilization under acidic conditions.^7^ GDPs are highly reactive substances that have direct cytotoxicity effects and form advanced glycation end products. The receptor for glycation end products activates key signal transduction pathways, including nuclear factor-kappa B, which induces fibrosis of the peritoneal membrane.^8^

In the 1990s, a second-generation double-chamber bag system was developed that separated the buffer from glucose, and thereby permitted the maintenance of a neutral-pH and reduced the formation of GDPs during heating and storage.^9^ Several previous studies have shown that the peritoneal membrane of adult patients undergoing PD using conventional fluids develops more severe fibrosis and vasculopathy and demonstrates higher levels of glycation end products accumulation from GDPs than that from patients undergoing PD using neutral-pH fluids.^10^^,^^11^ However, there have been no studies that have compared the peritoneal pathological changes in pediatric patients undergoing PD using conventional fluids or neutral-pH fluids. Furthermore, although a high glucose concentration has been reported to induce the production of fibrogenic growth factors in peritoneal mesothelial cells,^8^ the relationships between cumulative dialytic glucose exposure and peritoneal pathology has not been fully characterized.

Histomorphological changes have also been described in pediatric patients even after the short-term use of neutral-pH fluids for PD.^12^ Schaefer *et al.*^12^ described that early peritoneal inflammation, fibroblast activation, epithelial-mesenchymal transition and marked angiogenesis were observed in the peritoneal membrane samples obtained from children who had been undergoing PD using neutral-pH fluids for approximately a year. Parikova *et al.*^13^ suggested that low GDP neutral-pH fluids may not prevent but only reduce and retard the peritoneal alterations induced by continuous exposure to glucose-based dialysis fluids. However, the factors associated with the peritoneal pathology induced by the long-term use of neutral-pH fluids have not been identified.

In the present study, we aimed to compare the pathologic changes in the peritoneal membranes of pediatric patients undergoing PD using conventional fluids or neutral-pH fluids. We also aimed to identify the clinical factors, including cumulative dialytic glucose exposure, that are associated with the peritoneal pathology in pediatric patients who use neutral-pH fluids for more than 1 year. Furthermore, we aimed to elucidate the mechanisms of the pathological changes in the peritoneal membrane in such patients by performing an immunofluorescence (IF) study using markers of hypoxia, and angiogenesis.

## Methods

### Patients

**Figure 1:**
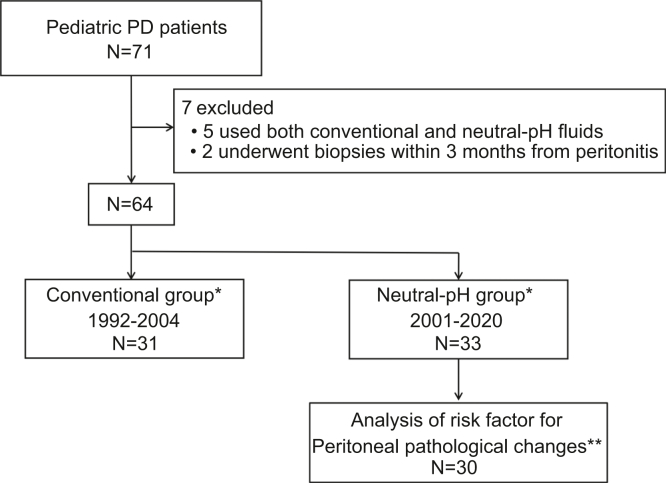
Study flowchart. Pediatric patients undergoing peritoneal dialysis (PD) using only conventional acidic fluids (conventional group) and those using only neutral-pH fluids (neutral-pH group) were compared. ∗Patients in the conventional group initiated PD between 1992 and 2004, and those in the neutral-pH group initiated PD between 2001 and 2020. ∗∗, Data of cumulative dialytic glucose exposure were available in 30 patients in the neutral-pH group. PD, peritoneal dialysis.

We performed a retrospective study of data obtained from medical records. Data relating to patients aged less than 15 years who commenced PD and underwent peritoneal membrane biopsy at our center between June 1992 and October 2020 were analyzed. The biopsy samples were obtained from the anterior abdominal wall. In our center, neutral-pH fluids have been used since 2001. Five patients who used both conventional and neutral-pH fluids between 2001 and 2004 were excluded from the study, as were 2 patients who underwent peritoneal membrane biopsy within 3 months of having peritonitis. After these exclusions, data from 31 patients who used only conventional fluids (conventional group) and 33 patients who used only neutral-pH fluids (neutral-pH group) were analyzed (Figure 1). The peritoneal pathologic changes were compared between the 2 groups, and then we determined whether these changes were associated with the duration of PD or cumulative dialytic glucose exposure in the neutral-pH group. In addition, the risk factors for peritoneal pathologic changes in the neutral-pH group were also identified. Cumulative glucose exposure by the dialysis solutions was assessed in the neutral pH group only. It was defined as the sum of the glucose concentrations of all instilled dialysis solutions between the initiation of PD and the peritoneal biopsy per body surface area. Finally, immunohistochemistry and immunofluorescence (IF) were performed to analyze the mechanisms of the peritoneal pathology.

### Processing of Peritoneal Membrane Samples

Peritoneal membrane samples were obtained using a previously described standard biopsy method.^6^ The samples were fixed in formalin and paraffin-embedded, then 3 μm-thick sections were prepared, dewaxed, rehydrated, and stained using hematoxylin and eosin or Masson’s trichrome. We used formalin and paraffin-embedded specimens, which are suitable for long-term storage with preserved morphology and reduced rates of oxidation.^14^

### Peritoneal Pathology

The peritoneal pathologic changes, such as thickening of the SMC zone, vasculopathy, new membrane formation, and greater submesothelial microvessel density were evaluated as previously reported.^6^^,^^10^^,^^12^ The thickness of the SMC zone was calculated as the mean value of the thickness measured at 5 points.^6^ The L/V ratio of the postcapillary venules was calculated for vessels with a diameter <50 μm, with the most severely affected postcapillary venules of each sample being selected for the analysis.^6^ New membrane formation was defined as the presence of an extra-thin fibrinous membrane that encapsulated the outer surface of the original peritoneal membrane.^15^^,^^16^ The submesothelial microvessel density was defined as the proportions of 0.2 mm^2^ areas of the SMC zone that were occupied by submesothelial microvessels. Vessels with an external diameter of <50 μm that included CD31 (monoclonal mouse antihuman CD31 antibody, JC70A, Dako, Denmark)-positive and podoplanin (monoclonal mouse antihuman podoplanin antibody, AngioBio, 11-003, CA)-negative endothelial cells were identified as submesothelial microvessels.^17^ The images were obtained by light microscopy at 200× magnifications using a BX-53 microscope equipped with a DP73 digital camera (Olympus, Tokyo, Japan) and evaluated using Image J software 1.51s (National Institutes of Health, Bethesda, MD). Peritoneal membrane samples obtained at the time of catheter insertion from patients who were initiating PD treatment served as controls.

### Immunohistochemical and Immunofluorescence Studies in the Neutral-pH Group

Antigen retrieval on formalin-fixed, paraffin-embedded samples was performed in Tris-EDTA buffer pH 7.8 in an autoclave at 125 °C for 5 minutes. The expression of HIF-1α, VEGF-α, transforming growth factor-β (TGF-β) and connective tissue growth factor (CTGF) were analyzed. Areas of immunoreactivity were identified using peroxidase/3,3′-Diaminobenzidine (DAB) immunohistochemistry with Histofine Simple Stain Rabbit Max Po (MULTI) (H1411, Nichirei, Japan) and Simple Stain DAB Substrate (H2102, Nichirei). Double IF staining for HIF-1α and VEGF-α were performed. Double IF staining for VEGF-α and cytokeratin, an epithelial marker, or α-smooth muscle actin, a myofibroblastic marker of epithelial-mesenchymal transition, were performed to analyze the origin of the VEGF-α-producing cells.

Double IF staining for VEGF-α and phosphorylated protein kinase B (Akt) or phosphorylated extracellular signal-regulated kinase (ERK) was also performed to investigate the mechanism of VEGF-α-production in the cells. Antibodies used for immunohistochemical and IF studies were listed in Supplementary Table S1 and S2.

### Cell Culture

We isolated HPMCs from human omentum as described previously.^18^ Harvesting of the omentum was permitted by the Medical Ethics Committee of Hiroshima Graduate School of Biomedical Science (E-2161). Written Informed consent was obtained from each patient. HPMCs were maintained in M199 medium (Lonza, Basel, Switzerland) containing 10% fetal bovine serum and penicillin-streptomycin. The cells were seeded into 6-well plates. At subconfluence, HPMCs were incubated for 24 hours in M199 medium and then treated with 4.25% high glucose or identical concentrations of mannitol for up to 48 hours. Mannitol was purchased from Fujifilm wako chemicals (Osaka, Japan) and glucose solution was purchased from Sigma-Aldrich (St. Louis, MO, USA).

### Western Blot

Western blot was performed as previously described.^19^ Briefly, total protein was extracted from cultured cells using Laemmili buffer (Sigma-Aldrich; St. Louis, MO, USA) and homogenized for 30 seconds using a VP-50 ultrasonic homogenizer (Taitec corp.; Saitama, Japan) at full power. Soluble proteins were further sonicated for 30 seconds 3 times. The concentrations of protein in the lysates were measured using a Pierce BCA protein assay kit (Thermo Fisher scientific; Waltham, MA, USA). An equal amount of each sample was analyzed by immunoblot analysis, as previously mentioned.^20^ The following primary antibodies were used: anti-HIF-1α antibody (1:1,000; 36,169; Cell Signaling); anti-VEGF-α antibody (1:1000; ab185238; abcam) and antiactin antibody (1:1,000; A2066; Sigma-Aldrich). The secondary antibodies were horseradish peroxidase-conjugated goat antirabbit and goat antimouse immunoglobulins (Dako, Glostrup, Denmark). Protein signals were detected using ImmunoStar LD (Fujifilm Wako Pure Chemical Corporation, Osaka, Japan) or Super Signal West Pico (Thermo Fisher Scientific) chemiluminescence reagents. The intensity of each band was analyzed using ImageJ software (National Institutes of Health; Bethesda, MD, USA).

### Statistics

Continuous datasets are shown as medians (interquartile ranges) and were compared using the Wilcoxon rank-sum test or analysis of variance, as appropriate. Categorical datasets were compared using Fisher’s exact test or Pearson’s test, as appropriate. To reduce the effects of confounding by indication, we performed propensity score matching of the data according to age at the time of peritoneal biopsy, sex, the duration of PD, cumulative dialytic glucose exposure, icodextrin use, and the number of episodes of peritonitis using logistic regression models. The conventional and neutral-pH groups were matched 1:1 using the logit of propensity score and a caliper width of 0.2 times the pooled standard deviation of the logit propensity scores. The standardized mean difference was used to assess the covariate balance between the 2 groups. A search of the published literature showed that a mean standardized difference of <0.20 was appropriate for the matching of the groups.^21^

Multivariable analysis was performed to identify the risk factors for peritoneal pathologic changes using a generalized linear model in the neutral pH group only. The selected variables were cumulative dialytic glucose exposure, the duration of PD, and icodextrin use. The independent variables were assessed for multicollinearity using a variance inflation factor of <5, and no collinearity was detected.

We used analysis of variance to evaluate the relationships between the proportions of the SMC zone that were HIF-1α, VEGF-α, TGF-β, and CTGF-positive respectively and the cumulative dialytic glucose exposure.

In the investigation of the expression of HIF-1α and VEGF-α in the cultured cell using Western blot, the data are summarized as mean ± SD for each group. Comparisons between 2 groups were tested using *t*-test.

Statistical analyses were performed using the JMP Pro 14.0.0 software package (SAS Institute Inc., Cary, NC) or R version 4.0.2 (The R Foundation, Vienna, Austria). The matching was performed using the matching package in R, and a *P* value of <0.05 was considered to indicate a statistically significant difference.

## Results

### Comparison of the Pathological Changes in the Peritoneal Membranes of Patients Using Conventional or Neutral-pH Fluids

Data for 31 patients in the conventional group and 33 in the neutral-pH group were analyzed (Figure 1). The clinical characteristics of the patients are shown in Table 1. There were no significant differences in age at the time of biopsy, sex, the duration of PD, cumulative dialytic glucose exposure, the incidence of peritonitis, or the timing of peritoneal biopsies between the 2 groups. Automated PD and continuous ambulatory PD were used in 21 and 10 patients, respectively, in the conventional group, while all patients underwent automated PD in the neutral-pH group. Icodextrin use was more prevalent in patients in the neutral-pH group (Table 1). Two patients in the conventional group developed encapsulating peritoneal sclerosis, whereas none of the patients in the neutral-pH group were diagnosed with encapsulating peritoneal sclerosis.

**Table 1:** Clinical characteristics of the patients at peritoneal biopsy

Clinical characteristics		Conventional group (*n* = 31)	Neutral-pH group (*n* = 33)	*P* value
Age at the time of peritoneal biopsy, yr		14 (8, 16)	11 (7, 17)	0.87^a^
Sex M : F		20 : 11	21 : 12	0.19^b^
Duration of PD, yrs		2.5 (1.3, 4.9)	3.5 (2.2, 5.3)	0.21^a^
Cumulative dialytic glucose exposure, kg/m^2^^c^		105.3 (36.9, 323.3)	187.6 (108.6, 299.9)	0.27^a^
PD modality (APD/CAPD)^d^		21/10	33/0	<0.01^b^
Use of icodextrin, cases (%)		0 (0)	12 (36)	<0.01^b^
Number of peritonitis, times		0 (0, 3)	1 (0, 2)	0.46^a^
The timing of peritoneal biopsies, cases (%)				
Catheter removal	Kidney transplantation	20 (65)	22 (67)	0.69^b^
	Switch to hemodialysis	6 (19)	5 (15)	
	Catheter obstruction	4 (13)	3 (9)	
Catheter insertion after graft loss		0 (0)	2 (6)	
Inguinal hernia surgery		1 (3)	1 (3)	

APD, automated peritoneal dialysis; CAPD, continuous ambulatory peritoneal dialysis; F, female; M, male; PD, peritoneal dialysis.

Continuous variables were shown in median (interquartile range).

a Mann-Whitney U test.

b Fisher's exact test.

c Data were available in 12 and 30 patients in the conventional and neutral-pH groups, respectively.

d A glucose-based long dwell was used in 16 and 17 APD patients in the conventional and the neutral-pH groups, respectively.

**Figure 2:**
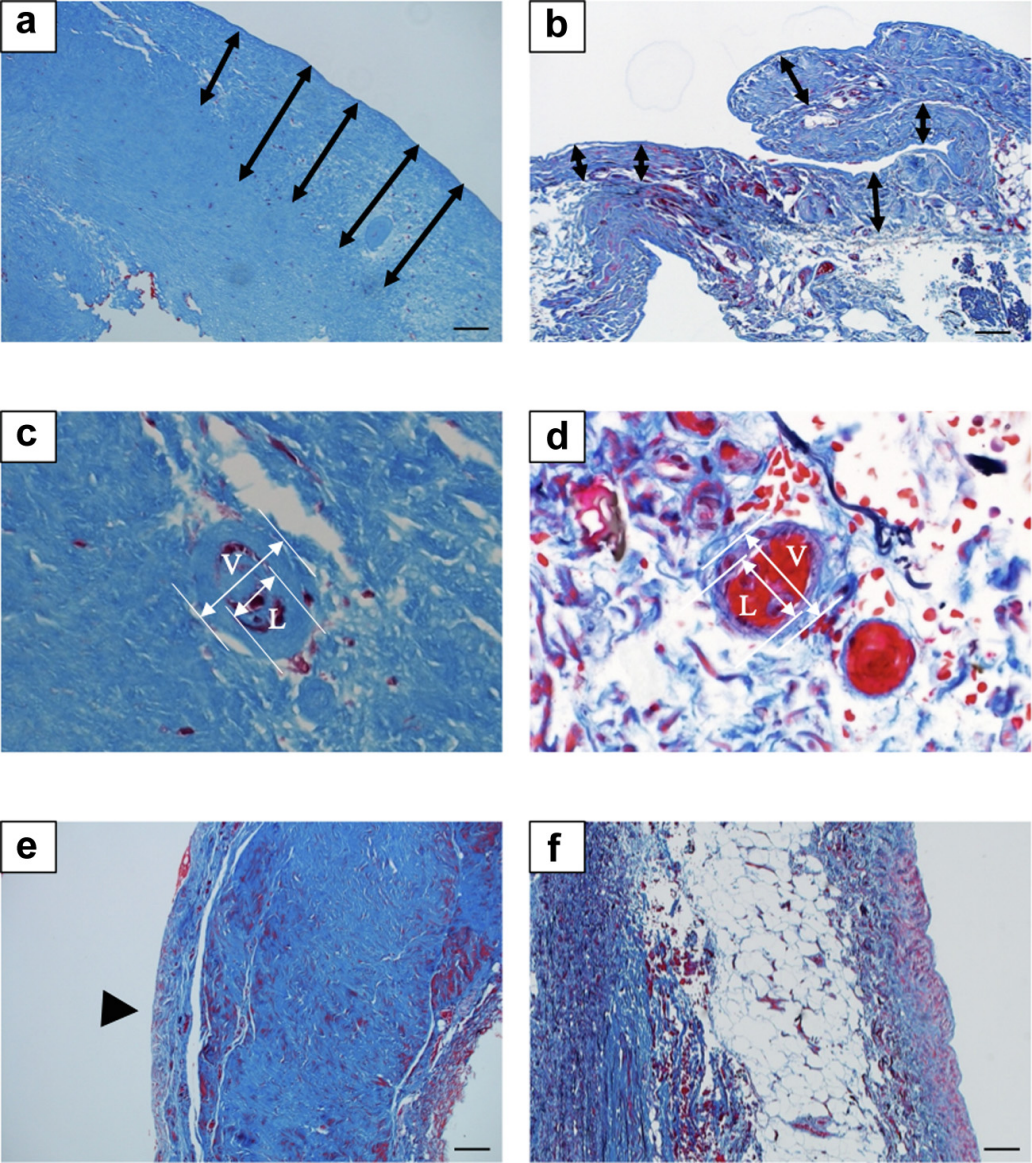
Pathologic changes in the peritoneal membrane of patients undergoing peritoneal dialysis. Representative images of sections of peritoneal membrane biopsy samples obtained from patients in (a, c, e) the conventional group and (b, d, f) the neutral-pH group. (a) Sample shows greater thickness of the submesothelial compact zone (arrows) than (b) sample. (c) Sample shows a lower L/V ratio than (d) sample. New membrane formation (arrowhead) was observed only in patients in the (compare samples e and f) conventional group. Masson’s trichrome staining; original magnifications, ×100. Scale bar = 100μm. L, luminal diameter; V, vessel diameter.

The propensity-score matching method yielded 19 well-balanced patient pairs with similar baseline clinical characteristics (Table 2). Histopathological analysis of samples from these patients revealed that the conventional group had a thicker SMC zone (*P* = 0.04, Figure 2a and 2b), a lower L/V ratio (*P* < 0.01, Figure 2c and 2d), and more frequent new membrane formation (*P* < 0.01, Figure 2e) than the neutral-pH group (Table 3). Indeed, there was no new membrane formation in the neutral-pH group (Figure 2f).

**Table 2:** Clinical characteristics of the patients after propensity score matching

Clinical characteristics	Conventional group (*n* = 19)	Neutral-pH group (*n* = 19)	*P* value
Age at the time of peritoneal biopsy, yr	10 (7, 15)	10 (6, 17)	0.91^a^
Sex (M : F)	12:7	11:8	0.74^b^
Duration of PD, yr	3.3 (1.3, 7.5)	4.5 (2.4, 5.8)	0.61^a^
Cumulative dialytic glucose exposure, kg/m^2^^c^	197.0 (37.1, 473.5)	157.6 (90.2, 291.4)	0.83^a^
PD modality (APD/CAPD)^d^	16/3	19/0	0.23^b^
Use of icodextrin, cases (%)	0 (0)	0 (0)	
Number of peritonitis, times	0 (0, 3)	1 (0, 2)	0.77^a^

APD, automated peritoneal dialysis; CAPD, continuous ambulatory peritoneal dialysis; F, female; M, male; PD, peritoneal dialysis.

Continuous variables were shown in median (interquartile range).

a Mann-Whitney U test.

b Fisher's exact test.

c Data were available in 8 and 16 patients in the conventional and neutral-pH groups, respectively.

d A glucose-based long dwell was used in 11 and 15 APD patients in the conventional and the neutral-pH groups, respectively.

**Table 3:** Comparison of the pathological changes between the conventional and neutral-pH groups

Pathological changes	Conventional group (*n* = 19)	Neutral-pH group (*n* = 19)	*P* value
Peritoneal thickness of the submesothelial compact zone, μm	454.5 (271.2, 598.9)	216.1 (142.3, 354.6)	0.04^a^
L/V ratio	0.54 (0.20, 0.60)	0.79 (0.70, 0.90)	<0.01^a^
New membrane formation, cases (%)	6 (32)	0 (0)	<0.01^b^

L/V ratio, luminal/vessel diameter ratio.

Continuous variables were shown in median (interquartile range).

a Mann-Whitney U test.

b Fisher's exact test.

### Relationships Between Peritoneal Pathological Changes and the Duration of PD in the Neutral-pH Group

**Figure 3:**
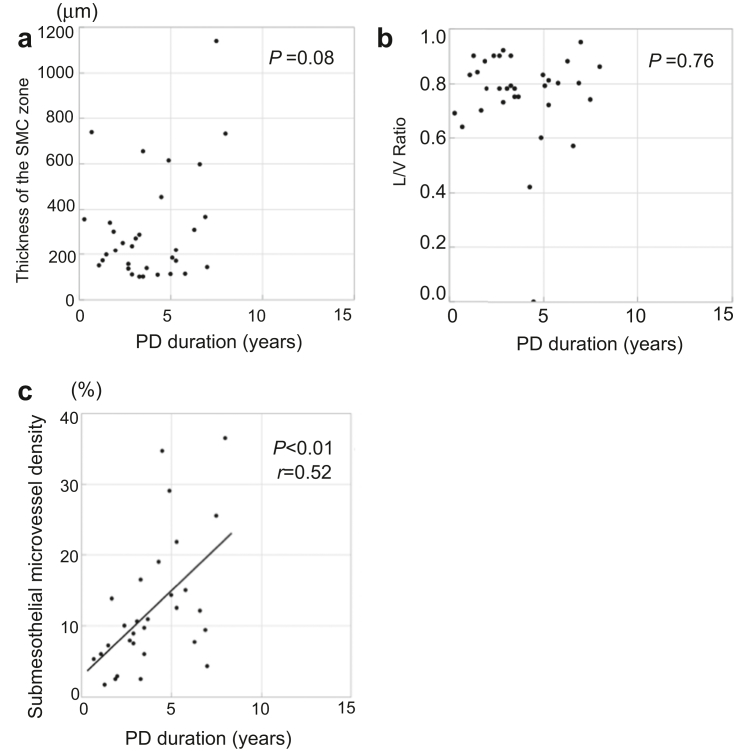
Relationships between the severity of the pathologic changes and the duration of PD in patients using neutral-pH fluids. The thickness of the (a) SMC zone and the (b) L/V ratio did not correlate with the duration of PD, but there was a significant correlation between submesothelial microvessel density and the (c) duration of PD. L/V, luminal diameter/vessel diameter; PD, peritoneal dialysis; SMC, submesothelial contact.

In the neutral-pH group, neither thickness of the SMC zone nor the L/V ratio correlated with the duration of PD (Figure 3a and 3b). However, the submesothelial microvessel density correlated with the duration of PD (*P* < 0.01, *r* = 0.52; Figure 3c).

### Relationships Between Peritoneal Pathological Changes and Cumulative Dialytic Glucose Exposure in the Neutral-pH Group

**Figure 4:**
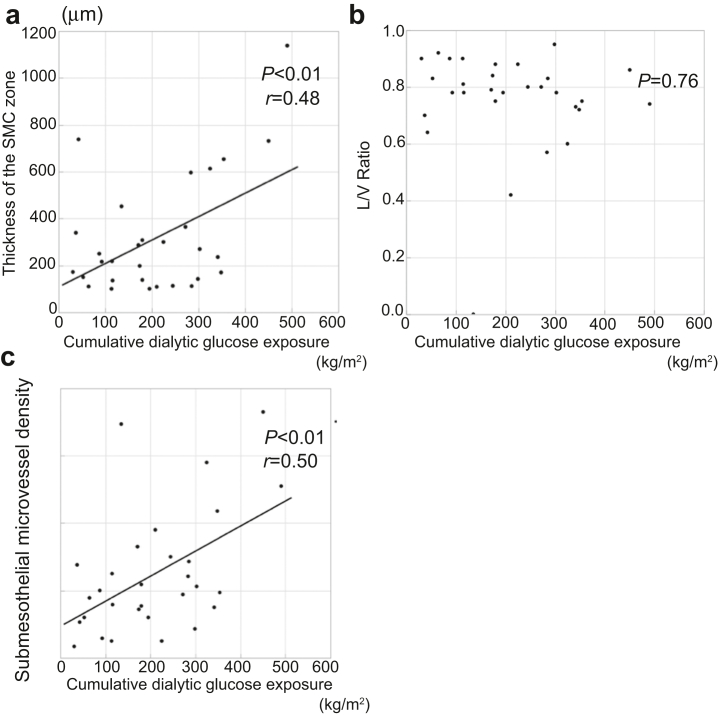
Relationships between the severity of the pathologic changes and cumulative dialytic glucose exposure in patients undergoing peritoneal dialysis using neutral-pH fluids. The thickness of the (a) SMC zone and the (c) submesothelial microvessel density correlated with the cumulative dialytic glucose exposure, but there was no significant correlation between the (b) L/V ratio and the cumulative dialytic glucose exposure. L/V, luminal diameter/vessel diameter; SMC, submesothelial compact.

In the neutral-pH group, the thickness of the SMC zone (*P* < 0.01, *r* = 0.48; Figure 4a) and the submesothelial microvessel density (*P* < 0.01, *r* = 0.50; Figure 4c) significantly correlated with cumulative dialytic glucose exposure. However, there was no significant correlation between the L/V ratio and the cumulative dialytic glucose exposure (Figure 4b).

### Risk Factors for Fibrosis and Angiogenesis in the Neutral-pH Group

To identify the risk factors for peritoneal fibrosis and angiogenesis in the neutral-pH group, we performed multivariate analysis using generalized linear models. This showed that cumulative dialytic glucose exposure was an independent risk factor for greater thickness of the SMC zone (OR, 1.54; 95% CI, 1.16–2.05) (Table 4) and higher submesothelial microvessel density (OR, 1.29; 95% CI, 1.01–1.64) (Table 5).

**Table 4:** Generalized linear model for increased thickness of the submesothelial compact zone: multivariable analysis

Variables	OR (95% CI)	*P* value
Duration of PD	1.00 (0.82, 1.22)	0.99
Cumulative dialytic glucose exposure	1.54 (1.16, 2.05)	<0.01
Use of icodextrin	0.91 (0.47, 1.76)	0.78

CI, Confidence interval; OR, Odds ratio; PD, peritoneal dialysis.

**Table 5:** Generalized linear model for increased submesothelial microvessel density: multivariable analysis

Variables	OR (95% CI)	*P* value
Duration of PD	1.02 (0.86, 1.22)	0.83
Cumulative dialytic glucose exposure	1.29 (1.00, 1.64)	0.04
Use of icodextrin	0.60 (0.31, 1.15)	0.12

CI, Confidence interval; OR, Odds ratio; PD, peritoneal dialysis.

### Cumulative Dialytic Glucose Exposure Correlates With the Area of HIF-1α Immunoreactivity in the SMC Zone, and HIF-1α Colocalizes with VEGF-α

**Figure 5:**
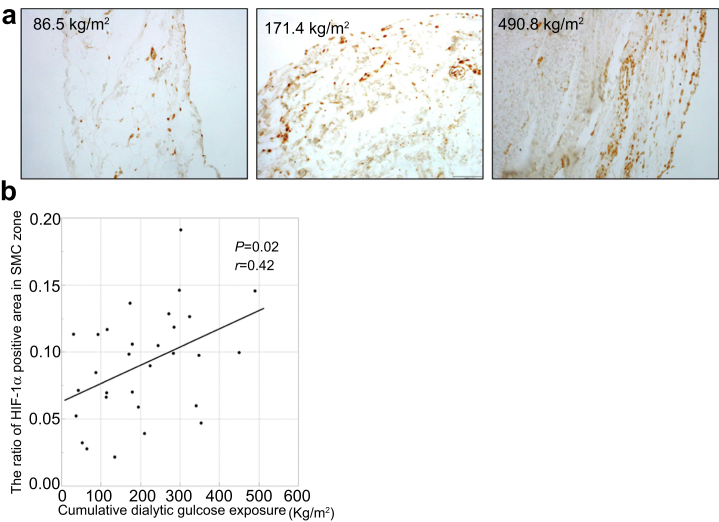
Relationship between the size of the HIF-1α-positive area and the cumulative dialytic glucose exposure in patients undergoing peritoneal dialysis using neutral-pH fluids. Representative images of immunohistochemical staining for HIF-1α in patients with (a) low, moderate, or high cumulative dialytic glucose exposure in the neutral-pH group. Original magnifications, ×200. There are larger HIF-1α-positive areas in patients with higher cumulative dialytic exposure. (b) The proportion of the submesothelial compact zone that was positive for HIF-1α significantly correlated with the cumulative dialytic glucose exposure. HIF-1α, hypoxia-inducible factor 1α. SMC, submesothelial compact.

**Figure 6:**
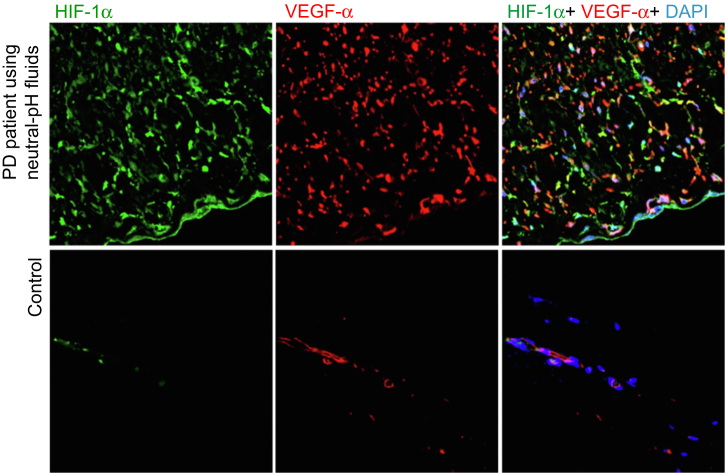
HIF-1α-producing cells show immunoreactivity for VEGF-α. A considerable number of cells show dual positive staining for HIF-1α and VEGF-α in the SMC zone. Original magnifications, ×400. HIF-1α, hypoxia-inducible factor 1α; PD, peritoneal dialysis; SMC, submesothelial compact; VEGF-α, vascular endothelial growth factor-α.

The results of an immunohistochemistry study of HIF-1α expression in the peritoneal membrane of patients using neutral-pH fluids are shown in Figure 5a. Patients with higher cumulative dialytic glucose exposure showed greater immunoreactivity of HIF-1α in the SMC zone of the peritoneal membrane (Figure 5a). The proportion of the SMC zone that was HIF-1α-positive significantly correlated with the cumulative dialytic glucose exposure (*r* = 0.42, *P* = 0.02; Figure 5b). Furthermore, double IF staining showed that a considerable number of cells in the SMC zone showed immunoreactivity for both HIF-1α and VEGF-α (Figure 6).

### Cumulative Dialytic Glucose Exposure Correlated With the Area of the SMC Zone that is VEGF-α-Positive in the Neutral-pH Group

**Figure 7:**
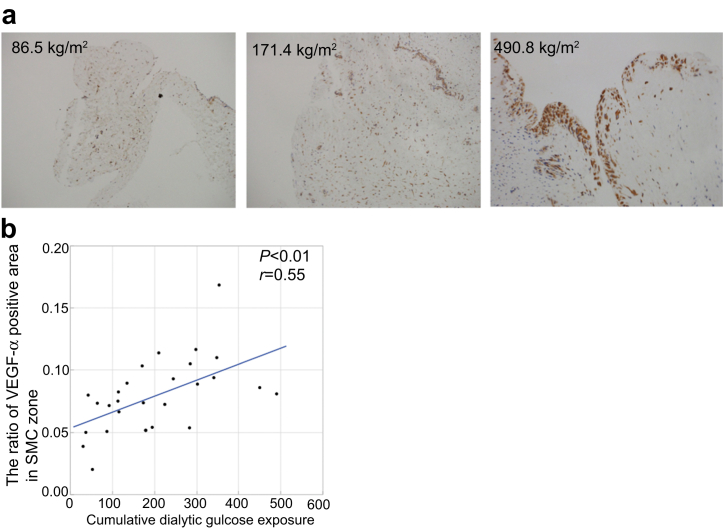
Relationships between the area of immunostaining for VEGF-α and the cumulative dialytic glucose exposure in patients undergoing peritoneal dialysis using neutral-pH fluids. (a) Representative images of immunohistochemical staining for VEGF-α in patients with low, moderate, or high cumulative dialytic glucose exposure in the neutral-pH group (a, original magnifications, ×200). (b) There are larger areas of VEGF-α immunoreactivity in patients with higher cumulative dialytic glucose exposure. The proportion of the area of the SMC zone that was VEGF-α-positive significantly correlated with the cumulative dialytic glucose exposure. SMC, submesothelial compact; VEGF-α, vascular endothelial growth factor-α.

The results of an immunohistochemistry study of VEGF-α expression in the peritoneal membrane of patients using neutral-pH fluids are shown in Figure 7. Patients with a larger cumulative dialytic glucose exposure showed greater immunoreactivity for VEGF-α in the SMC zone of the peritoneal membrane (Figure 7a), and the proportion of the SMC zone that was VEGF-α-positive significantly correlated with the cumulative dialytic glucose exposure (*r* = 0.55, *P* < 0.01; Figure 7b).

### Cumulative Dialytic Glucose Exposure Correlated With the Area of the SMC zone that is TGF-β-positive and CTGF-positive in the Neutral-pH Group

**Figure 8:**
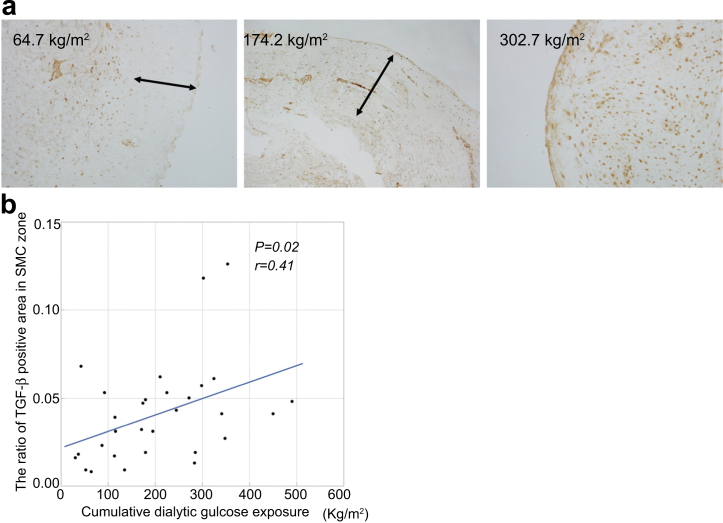
Relationships between the area of immunostaining for TGF-β and the cumulative dialytic glucose exposure in patients undergoing peritoneal dialysis using neutral-pH fluids. Representative images of immunohistochemical staining for TGF-β in patients with low, moderate, or high cumulative dialytic glucose exposure in the neutral-pH group (a, original magnifications, ×200). (a) There are larger areas of TGF-β immunoreactivity in patients with higher cumulative dialytic glucose exposure. Arrows indicate the submesothelial compact zone. (b) The proportion of the area of the submesothelial compact zone that was TGF-β-positive significantly correlated with the cumulative dialytic glucose exposure. SMC, submesothelial compact; TGF-β, transforming growth factor-β.

**Figure 9:**
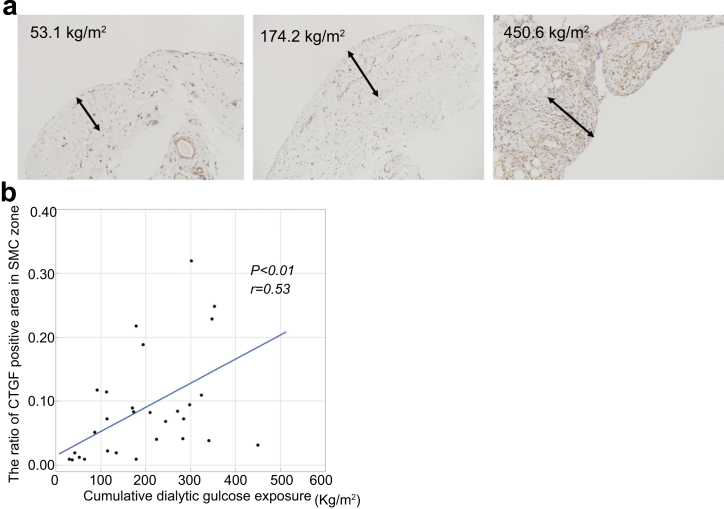
Relationships between the area of immunostaining for CTGF and the cumulative dialytic glucose exposure in patients undergoing peritoneal dialysis using neutral-pH fluids. Representative images of immunohistochemical staining for CTGF in patients with low, moderate, or high cumulative dialytic glucose exposure in the neutral-pH group (a, original magnifications, ×200). (a) There are larger areas of CTGF immunoreactivity in patients with higher cumulative dialytic glucose exposure. Arrows indicate the submesothelial compact zone. (b) The proportion of the area of the submesothelial compact zone that was CTGF-positive significantly correlated with the cumulative dialytic glucose exposure. CTGF, connective tissue growth factor; SMC, submesothelial compact; TGF-β, transforming growth factor-β.

immunohistochemistry studies of TGF-β and CTGF expressions in the peritoneal membrane of patients using neutral-pH fluids are shown in Figures 8 and 9. Patients with higher cumulative dialytic glucose exposure showed greater immunoreactivities of TGF-β and CTGF in the SMC zone of the peritoneal membrane (Figure 8a and 9a). The cumulative dialytic glucose exposure significantly correlated with the proportion of the SMC zones that was TGF-β-positive (*r* = 0.41, *P* = 0.02; Figure 8b) and CTGF-positive (*r* = 0.53, *P* < 0.01; Figure 9b).

### VEGF-α-producing Cells also Show Immunoreactivity for α-Smooth Muscle Actin and Cytokeratin

**Figure 10:**
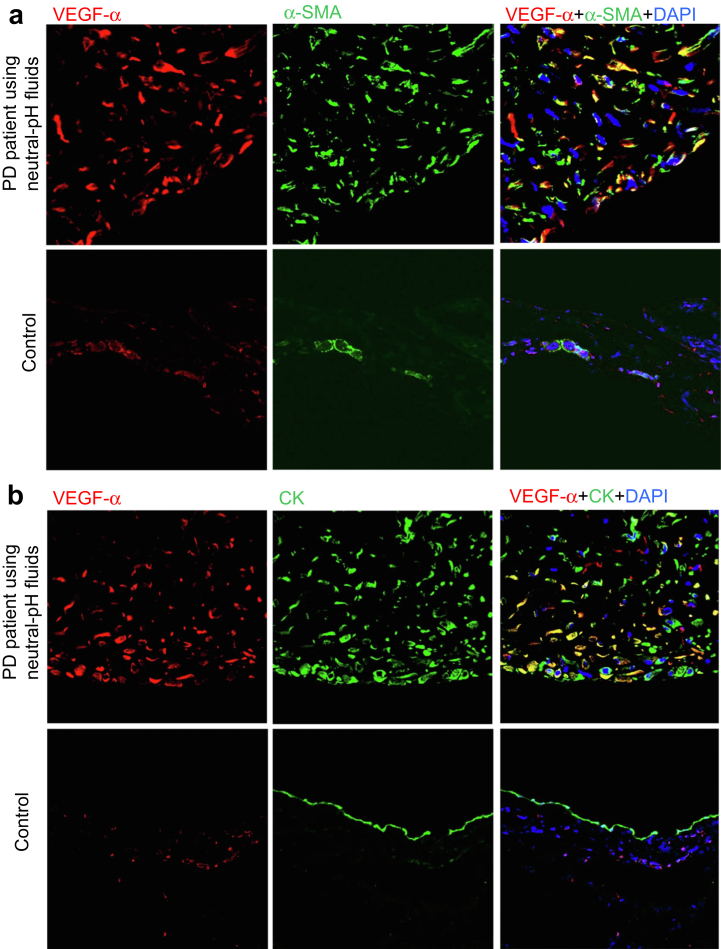
VEGF-α-producing cells also show immunoreactivity α-SMA and CK. A considerable number of vascular endothelial growth factor-α (VEGF-α)-producing cells showed staining for (a) α-SMA and (b) CK in the SMC zone. Original magnifications, ×400. α-SMA, α-smooth muscle actin; CK, cytokeratin; PD, peritoneal dialysis; SMC, submesothelial compact; VEGF-α, vascular endothelial growth factor-α.

An IF study showed that a considerable number of VEGF-α-producing cells in the SMC zone were also positive for α-smooth muscle actin (Figure 10a) and cytokeratin (Figure 10b) immunoreactivity.

### VEGF-α-producing Cells Show Phosphorylation of ERK and Akt

**Figure 11:**
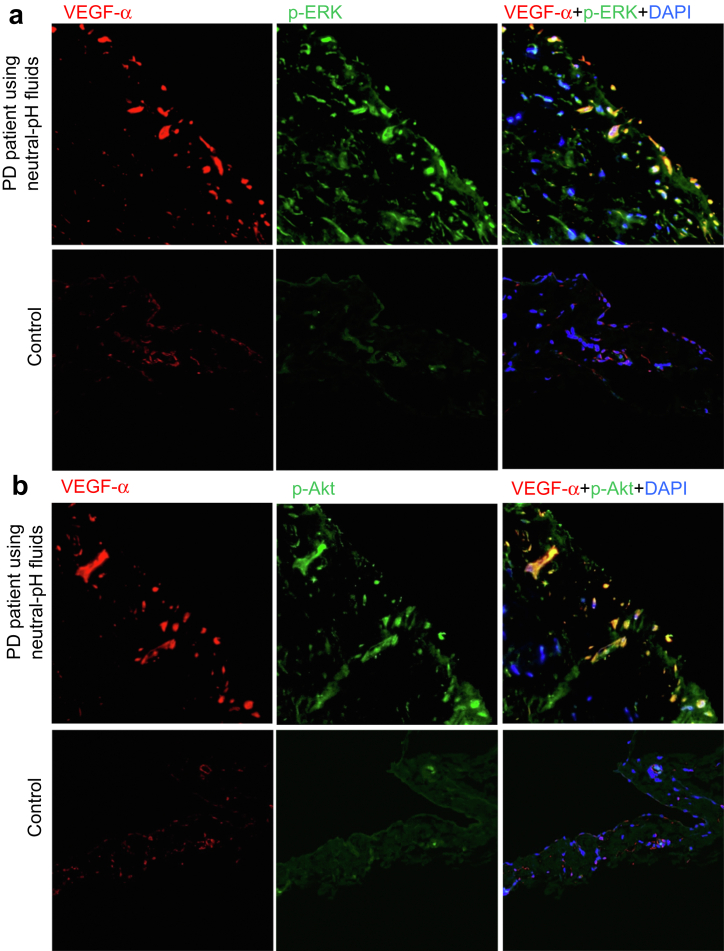
VEGF-α-producing cells also show phosphorylation of ERK and Akt. Most VEGF-α-producing cells were positive for the (a) phosphorylation of extracellular signal-regulated kinase (p-ERK) and (b) protein kinase B (p-Akt) in the SMC zone. Original magnifications, ×400. SMC, submesothelial compact; VEGF-α, vascular endothelial growth factor-α.

To further characterize the VEGF-α-producing cells, we determined whether signaling intermediates in pathways that are activated by exposure to high glucose concentrations were phosphorylated in these cells. IF study demonstrated that most of the VEGF-α-producing cells in the SMC zone contained phosphorylated extracellular signal-regulated kinase (Figure 11a) and phosphorylated protein kinase B (Figure 11b).

### High Glucose Significantly Increased both HIF-1α and VEGF-α Expression Levels in the Cultured Human Peritoneal Mesothelial Cells

**Figure 12:**
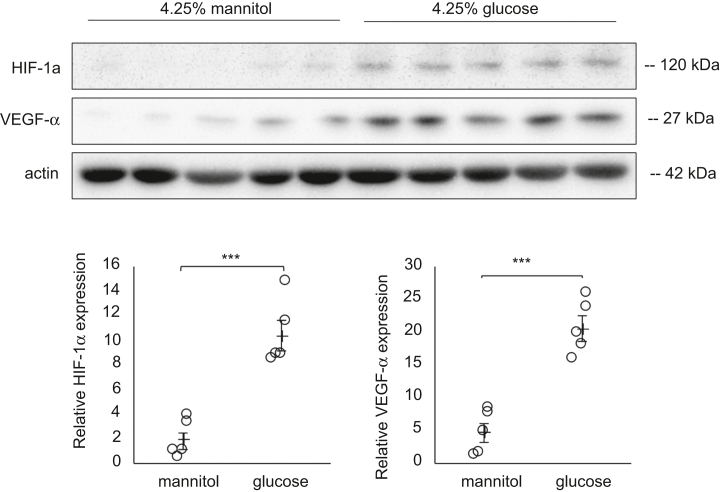
High glucose increased HIF-1α and VEGF-α expression. HPMCs were treated with 4.25% high glucose for 48 hours. High glucose upregulated HIF-1α and VEGF-α expression in HPMCs. (∗*P* < 0.001 *t*-test, *n* = 5 each group). ∗*P* < 0.001. HIF-1α, hypoxia-inducible factor 1α; VEGF-α, vascular endothelial growth factor-α.

To determine the mechanism by which glucose excess leads to HIF-1α activation in PD, we investigated HIF-1α and VEGF-α expression in high glucose condition using HPMCs. HPMCs were incubated with 4.25% high glucose for 48 hours. High glucose significantly increased both HIF-1α and VEGF-α protein expression levels compared to 4.25% mannitol control (Figure 12).

## Discussion

Pathological changes in the peritoneum of patients undergoing PD have been reported since the 1980s. Several previous studies have demonstrated that the uremic peritoneum gets progressively thicker, and that the L/V ratio decreases as the PD continues.^6^^,^^13^^,^^22^ Sherif *et al.* ^23^ reported that the number of intact vessels decreases with the duration of PD and that severe vasculopathy predominates after long-term PD. However, these studies were of patients using conventional fluids alone. Kawanishi *et al.*^11^ compared the peritoneal pathologic changes associated with conventional fluids (12 cases, 57.0 months of PD) and neutral-pH fluids (12 cases, 51.9 months of PD), and found that peritoneal membrane fibrosis and vasculopathy are significantly less marked in individuals using neutral-pH fluids than in those using conventional fluids. Tawada *et al.*^10^ reported similar findings, with the severity of vasculopathy correlating with the duration of PD in the conventional group but not in the neutral-pH group. Consistent with these findings, compared with neutral-pH fluids, we have shown for the first time that the use of conventional fluids was associated with greater thickness of the SMC zone, lower L/V ratio, and new membrane formation in pediatric patients undergoing PD.

Schaefer *et al.*^12^ showed that pediatric patients who had undergone PD using neutral-pH fluids for a median of 13 months had peritoneal membranes with higher microvessel density and submesothelial thickness. They also showed that these histologic changes were associated with the epithelial-mesenchymal transition and higher expression of profibrotic and proangiogenic cytokines. The present findings are consistent with those of Shaefer *et al.*,^12^ because the duration of PD significantly correlated with submesothelial microvessel density. However, in contrast, we found that the duration of PD did not significantly correlate with the thickness of the SMC zone, suggesting that a long-term PD may not cause serious peritoneal fibrosis if neutral-pH fluids are used.

New fibroblastic cells arise from the local conversion of mesothelial cells by epithelial-mesenchymal transition, which is induced by exposure to PD fluids, and they participate in inflammatory responses, extracellular matrix accumulation, and angiogenesis.^24^^,^^25^ Therefore, we hypothesized that high glucose exposure might be associated with worse peritoneal fibrosis and angiogenesis, and indeed, we found that cumulative dialytic glucose exposure significantly correlated with the severity of peritoneal fibrosis and angiogenesis. Furthermore, cumulative dialytic glucose exposure, but not the duration of PD, was an independent risk factor for greater thickness of the SMC zone and higher submesothelial microvessel density.

**Figure 13:**
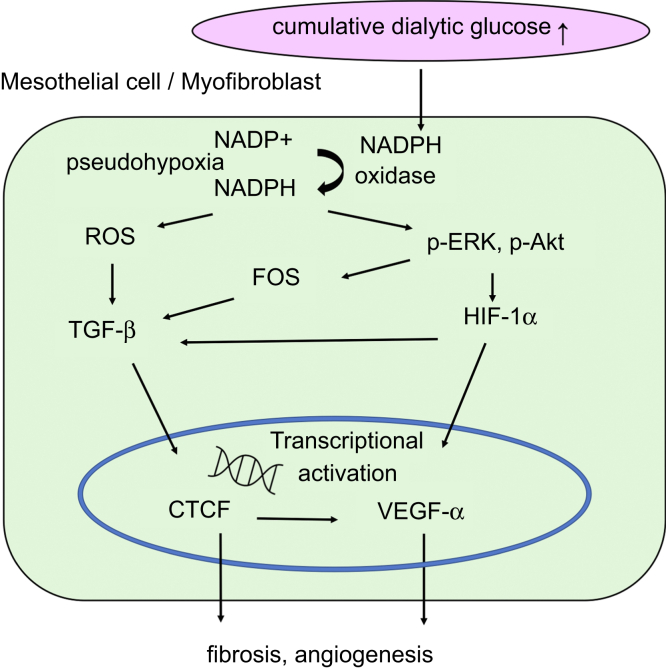
Schematic diagram of the potential mechanism whereby peritoneal fibrosis and angiogenesis are induced by a high cellular glucose load. A high cellular glucose load causes an increase in the intracellular NADH/NAD+ ratio, in a similar fashion to hypoxia. This pseudohypoxia induces ROS-mediated TGF-β expression and activates the ERK and Akt pathways, which stabilize HIF-1α. These processes promote CTGF and VEGF-α production, which causes peritoneal fibrosis and angiogenesis. Solid arrows, activation. CTGF, connective tissue growth factor; HIF-1α, hypoxia-inducible factor-1α; NAD+, nicotinamide adenine dinucleotide; NADH, nicotinamide adenine dinucleotide hydride; NADP, nicotinamide-adenine dinucleotide phosphate; p-Akt, phospho-protein kinase B; p-ERK, phospho-extracellular signal-related kinase; ROS, reactive oxygen species; TGF-β, transforming growth factor-β; VEGF-α, vascular endothelial growth factor-α.

A high cellular glucose load, like hypoxia, causes an increase in the intracellular nicotinamide adenine dinucleotide hydride/nicotinamide adenine dinucleotide ratio. This pseudohypoxia stimulates myofibroblasts to produce profibrotic and proangiogenic factors.^26^ In the present study, we have shown that the cumulative dialytic glucose exposure significantly correlates with immunoreactivity for HIF-1α, which increases the release of promoters of angiogenesis, such as VEGF-α.^27^ A considerable number of cells in the SMC zone showed immunoreactivity for both HIF-1α and VEGF-α. Furthermore, high glucose condition upregulated HIF-1α and VEGF-α protein expression in HPMCs. These findings suggest that even in patients using neutral-pH fluids, dialytic glucose exposure causes pseudohypoxia, which induces VEGF-α production (Figure 13).

We also sought to elucidate the mechanism of the peritoneal fibrosis and angiogenesis by assessing the expression of potential mediators using IF. The results of previous studies have suggested that increased TGF-β, CTGF and VEGF-α expression plays an important role in tissue fibrosis and angiogenesis.^12^^,^^24^^,^^26^^,^^28^, ^29^, ^30^ During this transitional process, mesothelial cells migrate from the superficial mesothelial layer toward the submesothelial area and produce extracellular matrix, which results in peritoneal fibrosis.^24^ It has also been shown that angiogenesis in the peritoneal membrane of patients using neutral-pH fluids is associated with high expression of VEGF-α.^12^ Under diabetic conditions, high glucose exposure has been reported to increase oxidative stress,^31^ which may play an important role in the development of fibrosis via the ERK and Akt pathways.^8^^,^^22^^,^^32^ In the neuronal cells, the phosphorylation of ERK and Akt, which results in HIF-1α stabilization and binding to DNA, leading to higher expression of VEGF-α.^33^ Both ERK and Akt are activated by high glucose concentrations, resulting in greater VEGF-α production in the vascular endothelial cells of the retinal fovea in patients with diabetic retinopathy and in human renal tubular epithelial cells.^34^^,^^35^ HIF-1α also stimulates other profibrotic factors like TGF-β and CTGF, which are known to be involved in the development of fibrosis.^36^ Consistent with these previous findings, we have shown that increased VEGF-α production in myofibroblasts in the submesothelial area of the peritoneum is involved in the mechanism of peritoneal fibrosis and angiogenesis. Taken together, our findings suggest that dialytic glucose exposure promotes the profibrotic process and causes pseudohypoxia in the peritoneal membrane, which induces VEGF-α production via the ERK and Akt pathways. (Figure 13)

The limitations of the present study include its retrospective nature and small sample size. Changes in glucose concentration in the peritoneal cavity per dwell time were not taken into consideration for the calculation of cumulative dialytic glucose exposure.^37^ In addition, the peritoneal pathologic findings were not assessed longitudinally in individual patients. Furthermore, the mRNA expression of markers of hypoxia and angiogenesis was not analyzed.

Finally, peritoneal function was not evaluated, because peritoneal equilibration tests were not performed at the same time as the peritoneal biopsies. Further studies are needed to determine whether the angiogenesis induced by neutral-pH fluids affects peritoneal functions, such as ultrafiltration and the diffusion of the substrate.

In conclusion, we have shown that pediatric patients undergoing PD using neutral-pH fluids show less severe deterioration of their peritoneal membrane than those using conventional fluids. However, neutral-pH fluids also have a detrimental effect on peritoneal membrane when glucose is overloaded as an osmotic substance, and cumulative dialytic glucose exposure is an independent risk factor for peritoneal fibrosis and angiogenesis in patients undergoing PD using neutral-pH fluids.

## Disclosure

All the authors declared no conflict of interest.

### Funding Information

This work was supported in part by Grants-in-Aid Scientific Research (C) [21K07829 to KM] from the Ministry of Education, Culture, Sports, Science and Technology of Japan.

### Ethical Approval

The study was approved by the Institutional Review Board at Tokyo Women’s Medical University (approval number 5336).

### Informed Consent

We applied opt-out method and the requirement for written informed consent was waived due to the retrospective nature of the study.

## Author Contributions

YS, KM, NK, KI, KH and MH designed the study; TI, KS and TM performed analysis using cultured cell; SK, KH and YY contributed to pathological diagnosis; YS and SH carried out experiments; DH performed statistical analysis. YS made the figures; YS, KM drafted the paper; KH and MH revised the paper; all authors approved the final version of the manuscript.

## Supplementary Material

Supplementary File (PDF)

**Table S1.** Immunohistochemistry antibodies

**Table S2.** Immunofluorescent antibodies

**STROBE Statement.**
