# Supplementary material for: Cumulative Dialytic Glucose Exposure is a Risk Factor for Peritoneal Fibrosis and Angiogenesis in Pediatric Patients Undergoing Peritoneal Dialysis Using Neutral-pH Fluids
Source: Kidney Int Rep. 2022 Sep 3;7(11):2431–45. doi: 10.1016/j.ekir.2022.08.013 (PMC9751577; doi:10.1016/j.ekir.2022.08.013)
Supplement: Supplementary File (PDF) [file mmc1.pdf]

Supplementary Table 1. Immunohistochemistry antibodies

No.	Antibody name	Clonality Species	Company	Product No.	Diluted
1	Anti-hypoxia inducible factor-1α antibody	rabbit polyclonal antibody	Thermo Fisher Scientific	BS-0737R	1:100
2	Anti- vascular endothelial growth factor-α antibody	rabbit monoclonal antibody	Abcam	EP1176Y	1:100
3	Anti-transforming growth factor-β antibody	rabbit monoclonal antibody	Abcam	EPR21143	1:100
4	Anti- connective tissue growth factor antibody	rabbit polyclonal antibody	Abcam	EPR20728	1:100

Supplementary Table 2. Immunofluorescent antibodies

No.	Antibody name	Clonality Species	Company	Product No.	Diluted
1	Anti-hypoxia inducible factor-1α antibody	rabbit polyclonal antibody	Thermo Fisher Scientific	BS-0737R	1:100
2	Anti- vascular endothelial growth factor-α antibody	rabbit monoclonal antibody	Abcam	EP1176Y	1:100
3	Anti- α -smooth muscle actin antibody	mouse monoclonal antibody	Agilent Dako	1A4	1:50
4	Anti-cytokeratin antibody	mouse monoclonal antibody	Sigma-Aldrich	C-11	1:50
5	Anti-p-AKT antibody	rabbit monoclonal antibody	Invitrogen	98H9L8	1:100
6	Anti- p-ERK antibody	rabbit monoclonal Antibody	Cell Signaling	9102	1:100
7	Anti-rabbit IgG, Superclonal recombinant secondary antibody (Alexa Fluor 568)	goat monoclonal antibody	Thermo Fisher Scientific	-	1:500
8	Anti-mouse IgG, Superclonal recombinant secondary (Alexa Fluor 488)	goat monoclonal antibody	Thermo Fisher Scientific	-	1:1000
9	Zenon Alexa Fluor 488 Rabbit IgG Labeling Kit	-	Thermo Fisher Scientific	Z25302	-

Modified STROBE Statement—checklist of items that should be included in reports of observational studies (Cohort/Cross-sectional and case-control studies)

	Item No	Recommendation	Page No	Relevant text from manuscript
**Title and abstract**	1	(*a*) Indicate the study's design with a commonly used term in the title or the abstract	1	
		(*b*) Provide in the abstract an informative and balanced summary of what was done and what was found	2	Line 52-59.
**Introduction**				
Background/rationale	2	Explain the scientific background and rationale for the investigation being reported	4-5	From page 4, line 6 to page 5, line 35.
Objectives	3	State specific objectives, including any prespecified hypotheses	6	Line 4-41.
**Methods**				
Study design	4	Present key elements of study design early in the paper	6	Line 4-41.
Setting	5	Describe the setting, locations, and relevant dates, including periods of recruitment, exposure, follow-up, and data collection	6-8	From page 6, line 4 to page 8, line 14.
Participants	6	(*a*) *Cohort study*—Give the eligibility criteria, and the sources and methods of selection of participants. Describe methods of follow-up<br><br>*Case-control study*—Give the eligibility criteria, and the sources and methods of case ascertainment and control selection. Give the rationale for the choice of cases and controls	6	Line 4-23.

		Cross-sectional study—Give the eligibility criteria, and the sources and methods of selection of participants		
Variables	7	Clearly define all outcomes, exposures, predictors, potential confounders, and effect modifiers. Give diagnostic criteria, if applicable	6-7	Page 6 line 31-41; page 7, line 1 to 29.
Data sources/ measurement	8*	For each variable of interest, give sources of data and details of methods of assessment (measurement).	6-8	Page 6, lines 3-11; From page 7, line 1 to page 8, line14.
Bias	9	Describe any efforts to address potential sources of bias	9-10	Page 9, Line 19 to to page 10, 14.
Study size	10	Explain how the study size was arrived at (if applicable)		
Quantitative variables	11	Explain how quantitative variables were handled in the analyses. If applicable, describe which groupings were chosen and why	7	Line 1-39.
Statistical methods	12	(*a*) Describe all statistical methods, including those used to control for confounding	9	From p9, line 19 to p10, Line 23.
		(*b*) Describe any methods used to examine subgroups and interactions	9	Line 46-56.
		(*c*) Explain how missing data were addressed		No missing data
		(*d*) *Cohort study*—If applicable, explain how loss to follow-up was addressed<br><br>*Case-control study*—If applicable, explain how matching of cases and controls was addressed	9-10	From p9, line 19 to p10, line 23.

		Cross-sectional study—If applicable, describe analytical methods taking account of sampling strategy		
		(*e*) Describe any sensitivity analyses		Not applicable.
**Results**				
Participants	13*	(a) Report numbers of individuals at each stage of study—eg numbers potentially eligible, examined for eligibility, confirmed eligible, included in the study, completing follow-up, and analyzed	10	Line 12-28.
		(c) **Use of a flow diagram**	Figure 1	
Descriptive data	14*	(a) Give characteristics of study participants (eg demographic, clinical, social) and information on exposures and potential confounders	10	Line 31-59.
		(b) Indicate number of participants with missing data for each variable of interest		Not applicable.
		(c) *Cohort study*—Summarise follow-up time (eg, average and total amount)		Not applicable.
Outcome data	15*	*Cohort study*—Report numbers of outcome events or summary measures over time		
		Case-control study—Report numbers in each exposure category, or summary measures of exposure		
		Cross-sectional study—Report numbers of outcome events or summary measures	Table 1-3	

Main results	16	(*a*) Give unadjusted estimates and, if applicable, confounder-adjusted estimates and their precision (eg, 95% confidence interval). Make clear which confounders were adjusted for and why they were included	Table 4-5	
Other analyses	17	Report other analyses done—eg analyses of subgroups and interactions, and sensitivity analyses		Not applicable.
**Discussion**				
Key results	18	Summarise key results with reference to study objectives	16	Line 28-41.
Limitations	19	Discuss limitations of the study, taking into account sources of potential bias or imprecision. Discuss both direction and magnitude of any potential bias	16	Line 6-17.
Interpretation	20	Give a cautious overall interpretation of results considering objectives, limitations, multiplicity of analyses, results from similar studies, and other relevant evidence	13-16	From page 13, line 43 to page 16, line 5.
Generalisability	21	Discuss the generalisability (external validity) of the study results	16	Line 7-17.

*Give information separately for cases and controls in case-control studies and, if applicable, for exposed and unexposed groups in cohort and cross-sectional studies.

Note: An Explanation and Elaboration article discusses each checklist item and gives methodological background and published examples of transparent reporting. The STROBE checklist is best used in conjunction with this article (freely available on the Web sites of PLoS Medicine at http://www.plosmedicine.org/, Annals of Internal Medicine at http://www.annals.org/, and Epidemiology at http://www.epidem.com/). Information on the STROBE Initiative is available at www.strobe-statement.org.
